# The effect of motivational determinants on elite Wrestlers’ ıntentions to continue in sport: The mediating role of enjoyment

**DOI:** 10.1371/journal.pone.0353067

**Published:** 2026-07-10

**Authors:** Burhan Özkurt, Ersin Eskiler

**Affiliations:** 1 Faculty of Sports Sciences, Sivas Cumhuriyet University, Sivas, Türkiye; 2 Sakarya University of Applied Sciences, Sakarya, Türkiye; Çanakkale Onsekiz Mart University: Canakkale Onsekiz Mart Universitesi, TÜRKIYE

## Abstract

This study aimed to examine the associations of motivational determinants, including goal orientation, satisfaction of basic psychological needs, and motivation, with elite wrestlers’ intention to continue in sport, and to evaluate the indirect role of enjoyment in these associations. An explanatory sequential mixed-methods design was used. The quantitative sample comprised 374 elite wrestlers competing under the Turkish Wrestling Federation, and the qualitative sample comprised 16 volunteer elite wrestlers competing in freestyle and Greco-Roman wrestling. Quantitative data were collected using validated scales measuring goal orientation, basic psychological need satisfaction, sport motivation, enjoyment, and intention to continue in sport, and were analyzed using descriptive statistics and structural equation modeling through SPSS and AMOS. Qualitative data were collected through a semi-structured interview form and analyzed using content and descriptive analysis techniques in MAXQDA 2020. The quantitative findings showed that goal orientation, satisfaction of basic psychological needs, motivation, and enjoyment had significant direct, indirect, and total associations with intention to continue in sport. Enjoyment showed an indirect role in these associations, and the proposed model explained 41% of the variance in intention to continue in sport. The qualitative findings were classified under the theme “Factors Associated with Intention to Continue in Sport” and grouped into positive and negative factors with internal and external subcategories. Internal positive factors included commitment and passion, achievement goals, and enjoyment, whereas external positive factors included reference groups, coach interaction, and social support. Negative factors included failure, negative psychological processes, injustice, sport-specific difficulties, and lack of resources. Overall, the findings indicate that elite wrestlers’ intention to continue in sport is associated with motivational, emotional, social, and contextual factors. The qualitative findings further suggest that these factors are reflected in athletes’ experiences regarding sport continuation.

## Introduction

Elite athletic participation is often associated from an external perspective with success, prestige, and high performance; however, behind this process lies a challenging career accompanied by intense physical and psychological demands. Athletes are engaged in a multidimensional process that requires long-term training loads, entails the risk of injury, involves coping with failure, burnout, and continuous self-sacrifice [1,2]. The sustainability of this process depends not only on physical capacity but also on the athlete’s ability to cope with stress, maintain motivation, and sustain psychological resilience [3,4]. Therefore, examining the psychological processes that support elite athletes’ determination to continue in sport despite these challenges is important for understanding long-term sport participation and performance continuity.

At the elite level, the determination to continue in sport depends not only on maintaining physical capacity but also on how athletes’ motivational, cognitive, and emotional processes operate together [5–7]. In this context, how athletes define success, the extent to which their basic psychological needs are satisfied, the motivational sources that guide their behavior, and the level of enjoyment derived from sport emerge as salient psychological factors in understanding the intention to continue in sport. Although these variables are important in their own right, they need to be addressed in mutual interaction and within a holistic framework in order to comprehensively explain elite wrestlers’ intention to continue in sport.

One of the fundamental approaches explaining motivational processes in sport is Achievement Goal Theory [8]. This theory suggests that how individuals define success shapes their behaviors and their ways of coping with failure in line with their goal orientations [9,10]. Athletes’ orientations toward achievement are generally addressed in two dimensions: task orientation and ego orientation [11,12]. While task orientation focuses on personal development, learning, and effort, ego orientation evaluates success through comparison with others and establishing superiority. Therefore, goal orientation is regarded as an important cognitive–motivational variable that structures athletes’ perceptions of success and plays a role in shaping their intention to continue in sport [13,14].

Another fundamental construct that sustains athletes’ behaviors is the satisfaction of basic psychological needs as explained within the framework of Self-Determination Theory [15]. According to this theory, the fulfillment of the needs for autonomy, competence, and relatedness supports individuals’ intrinsic motivation and psychological well-being. In the sport context, satisfying these needs enables athletes to feel competent, valued, and socially connected, thereby strengthening their commitment to sport [16,17]. In contrast, insufficient satisfaction of these needs is associated with decreased motivation, burnout, and a tendency to withdraw from sport. Therefore, particularly in disciplines such as wrestling that involve high physical and psychological demands, the satisfaction of basic psychological needs is considered an important variable in explaining athletes’ intention to continue in sport [7].

One of the key determinants of sport participation and continuation is the direction and source of motivation [18]. Motivation is defined as a dynamic process that initiates, directs, and sustains individuals’ behaviors [19,20]. In this process, intrinsic motivation stems from individuals’ finding the activity meaningful and enjoyable, whereas extrinsic motivation is based on environmental factors such as rewards, recognition, and social approval [15,21]. In elite athletes, supporting these two types of motivation in a balanced manner plays a decisive role not only in the continuity of performance but also in the sustainability of the intention to continue in sport [22,23].

One of the key psychological factors associated with elite wrestlers’ intention to continue in sport is the enjoyment derived from sport [7]. Enjoyment refers to the positive emotional state experienced by individuals during sport participation and is considered an important intrinsic resource that supports the continuity of sport involvement [15,24–26]. According to the Sport Commitment Model, enjoyment is one of the strongest determinants of maintaining participation in sport activities [27]. This feeling not only enhances commitment to sport but also facilitates coping with fatigue and stress, thereby contributing to the maintenance of the behavior [28,29]. In addition, enjoyment derived from sport is accepted as an important component of the psychological process underlying the intention to continue in sport [7,30]. Athletes’ tendency to maintain an activity is closely related not only to their desire to achieve their goals but also to how they experience that activity. Perceiving the sport experience as enjoyable, meaningful, and satisfying strengthens athletes’ determination to sustain this behavior.

The intention to continue in sport is considered a fundamental psychological construct that refers to an individual’s conscious tendency, determination, and desire to maintain sport participation in the future [7,31]. According to the Theory of Planned Behavior, this intention is shaped by individuals’ attitudes toward sport, subjective norms reflecting the expectations of their social environment, and perceived behavioral control regarding performing the behavior [32,33]. In the sport context, these components constitute the cognitive basis of individuals’ tendencies to sustain participation behavior [34,35].

However, the intention to continue in sport is not limited to cognitive evaluations alone; processes such as the satisfaction of basic psychological needs, motivational regulations, and enjoyment derived from sport strengthen this intention [36,37]. Therefore, in order to explain the intention to continue in sport, the psychological processes that shape this intention need to be considered together. This necessity becomes even more evident particularly in challenging and competitive disciplines such as wrestling, which require not only high levels of physical endurance but also the capacity to cope with intense psychological pressures [38,39]. For this reason, understanding these psychological processes in elite wrestlers is also important in terms of performance sustainability and preventing disengagement from sport [7].

In the existing literature, variables such as goal orientation, satisfaction of basic psychological needs, motivation, enjoyment, and the intention to continue in sport are mostly examined either separately or within models that consider a limited number of variables together. These studies generally focus on one of the approaches such as Achievement Goal Theory, Self-Determination Theory, or the Theory of Planned Behavior, and attempts to address different theoretical frameworks in an integrated manner remain limited [37,40]. In particular, the limited number of studies examining the mediating role of enjoyment leads to an insufficient explanation of the psychological mechanisms underlying the intention to continue in sport [28,30]. Moreover, the scarcity of scale-based structural model studies conducted with elite wrestlers indicates a significant empirical gap in this area.

This study proposes a model that integrates different theoretical approaches by taking these limitations into account. The aim of the study is to examine the effects of goal orientation, satisfaction of basic psychological needs, and motivation level on the intention to continue in sport among elite freestyle and Greco-Roman wrestlers, and to test the mediating role of enjoyment in this relationship. The study was structured using a mixed-method approach; the proposed structural model was tested with quantitative data, and the findings were supported and elaborated with qualitative data in line with athletes’ experiences. This model is based on the core assumptions of Achievement Goal Theory [8], Self-Determination Theory [15], the Theory of Planned Behavior [32], and the Sport Commitment Model [27]. In this respect, the study aims to contribute to the literature by addressing, within a holistic framework, the psychological processes that explain elite wrestlers’ continuation in sport. In line with the model proposed in this study, the following hypotheses were tested:

**H**_**1**_: Goal orientation is positively associated with basic psychological need satisfaction.

**H**_**2**_: Goal orientation is positively associated with motivation.

**H**_**3**_: Basic psychological need satisfaction is positively associated with motivation.

**H**_**4**_: Basic psychological need satisfaction is positively associated with enjoyment.

**H**_**5**_: Motivation is positively associated with enjoyment.

**H**_**6**_: Motivation is positively associated with intention to continue in sport.

**H**_**7**_: Enjoyment is positively associated with intention to continue in sport.

## Methods

In this section, the research design, study group, data collection instruments, and the methods used for data collection and analysis are described.

### Research model

**Fig 1:**
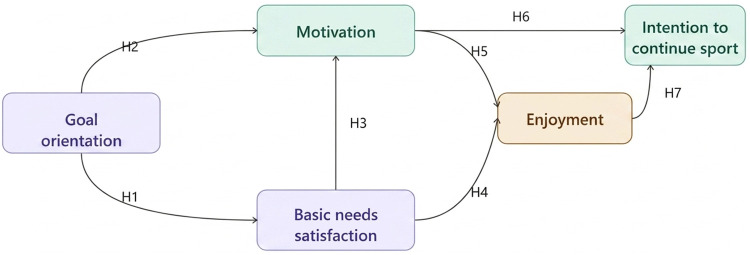
The structural model of the study.

This study was structured using a mixed-method approach to examine the relationships among goal orientation, satisfaction of basic psychological needs, motivation, enjoyment, and the intention to continue in sport among elite wrestlers. In the quantitative phase, the structural model developed on the basis of theoretical foundations was tested; in the qualitative phase, the quantitative findings were deepened in line with athletes’ experiences. In the structural model, the direct and indirect structural associations between goal orientation, satisfaction of basic psychological needs, motivation, and intention to continue in sport were examined, and the indirect role of enjoyment in these relationships was evaluated. Structural Equation Modeling was used to test the model, and the analyses were conducted using AMOS software. The tested model is presented in Fig 1.

### Research design

This study was conducted using an explanatory sequential mixed-methods design. In the first phase, quantitative data were collected, and the associations among goal orientation, satisfaction of basic psychological needs, motivation, enjoyment, and intention to continue in sport were tested using the structural model. In the second phase, qualitative data were collected to explain and deepen the quantitative findings [41]. The quantitative phase of the study was structured within a correlational survey model, whereas the qualitative phase was based on a phenomenological design. Due to the cross-sectional and correlational nature of the quantitative phase, the coefficients obtained in the structural model were interpreted as structural associations among the variables rather than causal effects. The phenomenological design is a qualitative approach that enables the meanings participants attribute to their experiences regarding a particular phenomenon to be revealed [42]. This design was preferred to explain the psychological, social, and emotional processes underlying the associations identified through quantitative analyses, based on the experiences of elite wrestlers.

### Participants and research group

#### Quantitative study group.

The quantitative dataset consisted of 374 elite wrestlers determined through purposive sampling. The sample comprised 325 men (86.9%) and 49 women (13.1%), aged 17–35 years. Participants were selected from freestyle and Greco-Roman wrestlers actively competing in the Junior, U23, and Senior categories affiliated with the Turkish Wrestling Federation. The inclusion criteria were being an actively licensed wrestler, competing in one of the relevant age categories, and having national or international competition experience within the last four years.

Of the participants, 41.2% were aged 17–19, 37.2% were aged 20–23, and 21.7% were aged 24 years or older. Regarding wrestling style, 50.3% competed in Greco-Roman and 49.7% in freestyle. In terms of sport experience, 18.7% had 4–6 years, 37.2% had 7–9 years, and 44.1% had 10 years or more of sport experience. According to competition category, 48.7% competed in Juniors, 29.7% in U23, and 21.7% in Seniors. Participants had varying competitive achievements at the Turkish Championship, international tournament, European Championship, World Championship, Mediterranean Games, and Olympic Games levels.

An a priori power analysis was conducted based on the RMSEA approach in structural equation modeling to evaluate the adequacy of the quantitative sample size. In the analysis, RMSEA = .08 was specified to represent a medium degree of model misfit, with an alpha level of α = .05 and a target power of 1 − β = .80. The analysis yielded a Type II error probability of β = .194 and an achieved power of.806. In addition, the A–B ratio was.258 and the noncentrality parameter (NCP) was 74.20. These results indicate that the quantitative sample of 374 participants had sufficient statistical power to test the proposed structural model [43,44].

#### Qualitative study group.

A total of 16 elite wrestlers voluntarily participated in the qualitative phase of the study. The qualitative sample comprised 14 men (87.5%) and 2 women (12.5%), with ages ranging from 20 to 33 years. Regarding wrestling style, 8 participants competed in freestyle (50%) and 8 in Greco-Roman (50%). The participants had 11 years to 20 years or more of sport experience and consisted of elite athletes with national and international competitive experience. In terms of the highest level of sporting achievement, 12.5% had placed at the World University Championships, 18.75% at the Junior European Championships, 12.5% at the Junior World Championships, 6.25% at the U23 European Championships, 31.25% at the Senior World Championships, and 18.75% at the Olympic Games.

Participants were determined using purposive sampling and were selected from among elite wrestlers who could provide in-depth information appropriate to the purpose of the study [45]. In phenomenological research, sample size is evaluated primarily on the basis of participants’ experiential richness regarding the phenomenon under investigation and the achievement of data saturation rather than statistical representativeness [41]. In this study, the codes, categories, and themes emerging during the interviews were monitored; no new theme or subcategory emerged from the 14th participant onward in the interviews with elite wrestlers. Therefore, thematic saturation was considered to have been achieved. Two additional interviews were conducted to confirm saturation, strengthen thematic diversity, and enhance the consistency of the findings. Thus, the data obtained from a total of 16 participants were considered sufficient to explain the phenomenon under investigation.

### Data collection ınstruments

The data collection instruments used in this study consisted of quantitative and qualitative tools. In the quantitative phase, scales were used to measure the variables included in the structural model of the study, whereas in the qualitative phase, a semi-structured interview form was employed.

### Quantitative data collection ınstruments

In this study, the construct validity of all quantitative measurement instruments was evaluated using confirmatory factor analysis (CFA), and internal consistency was assessed through Cronbach’s alpha (α) and McDonald’s omega (ω) coefficients. The CFA results indicated that each scale demonstrated acceptable fit indices in the sample of elite wrestlers (χ²/df < 3, CFI > .90, RMSEA < .08). During the CFA procedures, some items were removed due to low factor loadings (<.40) or because they weakened model fit, and the final factor structure of each scale was established. The quantitative data collection instruments used in the study are as follows:

Personal Information Form. The Personal Information Form, developed by the researcher to determine participants’ demographic characteristics, includes basic information such as gender, age, wrestling style, sport experience, and educational status.

Exercise Goal Orientation Questionnaire (EGOQ). The Exercise Goal Orientation Questionnaire was developed by Petherick and Markland and adapted into Turkish by Ersöz and colleagues [46,47]. The scale consists of 10 items rated on a 5-point Likert scale and includes two subdimensions: task orientation and ego orientation.

Basic Psychological Need Satisfaction in Sport Scale (BPNS-Sport). The Basic Psychological Need Satisfaction in Sport Scale was developed by Ng and colleagues and adapted into Turkish by Gümüşay and Argan [48,49]. The scale consists of 14 items rated on a 5-point Likert scale and includes three subdimensions: competence, autonomy, and relatedness.

**Sport Motivation Scale-II (SMS-II).** To determine participants’ levels of motivation toward sport, the Sport Motivation Scale-II, developed by Pelletier and colleagues and adapted into Turkish by Yıldız and colleagues, was used [50,51]. Developed based on Self-Determination Theory, the scale consists of 16 items rated on a 7-point Likert scale and includes six subdimensions: intrinsic regulation, integrated regulation, identified regulation, introjected regulation, external regulation, and amotivation. In this study, the measurement structure of the scale was evaluated using confirmatory factor analysis. In the initial analyses, some items were found to have low factor loadings (<.40) and to weaken the fit indices of the measurement model. Therefore, these items were removed from the model. In the final structural equation model, the motivation variable was represented by the remaining 9 items, which retained theoretically meaningful indicators of sport motivation within the framework of Self-Determination Theory.

**Physical Activity Enjoyment Scale (PACES).** To assess participants’ enjoyment derived from their physical activity and sport experiences, the Physical Activity Enjoyment Scale, developed by Mullen and colleagues and adapted into Turkish by Özkurt and colleagues, was used [25,52]. The scale consists of 8 items rated on a 7-point Likert scale. In this study, the measurement structure of the scale was examined using confirmatory factor analysis, and three items with low factor loadings (<.40) that weakened model fit were excluded from the analysis. In the final structural model, the enjoyment variable was represented by 5 items that demonstrated acceptable factor loadings and fit indices.

**Intention to Continue Sport Scale.** The Intention to Continue Sport Scale was developed by Özkurt and was validated and introduced to the literature by Özkurt and colleagues [7,31]. Developed based on the Theory of Planned Behavior, the scale measures individuals’ levels of intention to continue participating in sport. The scale consists of 6 items rated on a 5-point Likert scale and has a single-factor structure. Although the original form comprises 6 items and one dimension, the confirmatory factor analysis conducted in this study indicated that two items had low factor loadings (<.40) and negatively affected model fit. Therefore, a single-factor version with 4 items was used in the analyses.

### Qualitative data collection ınstruments

In the qualitative phase of the study, a semi-structured interview form was used to gain an in-depth understanding of elite wrestlers’ perceptions and experiences regarding their intention to continue in sport. Voluntary consent was obtained from all participants, all interviews were audio-recorded, and the recordings were transcribed verbatim.

### Semi-structured ınterview form

In the qualitative phase of the study, a semi-structured interview form was employed to examine elite wrestlers’ perceptions and experiences related to their intention to continue in sport in depth. The form was developed based on the relevant literature and the opinions of three field experts. Initially, a draft form consisting of 15 questions was evaluated in terms of content appropriateness and alignment with the purpose of the study and was reduced to six core questions. To assess the applicability of the form, pilot interviews were conducted with four elite wrestlers; based on the feedback received, the wording and order of the questions were revised. The draft and final versions of the semi-structured interview form are presented in S3 File.

### Researcher role and ethical principles

The researcher has extensive field experience in wrestling as an elite athlete and coach for many years and conducts academic studies in the field of sport psychology. This experience contributed to understanding the research context and establishing effective communication with participants during the qualitative data collection process. Nevertheless, to minimize potential biases that may arise from the researcher’s close involvement with the field, the interviews were conducted in line with the semi-structured interview form; during the analysis process, the researchers adhered to participants’ statements and evaluated the data through systematic coding.

The study was conducted in accordance with ethical principles, and ethical approval was obtained from the Ethics Committee of the Graduate Education Institute Directorate of Sakarya University of Applied Sciences, with the decision dated 21.05.2021 and numbered E-26428519-044-12077. Prior to data collection, all participants were informed about the purpose and scope of the study, the procedures to be implemented, the principles of voluntariness and confidentiality, and their right to withdraw from the study at any time. Written informed consent was obtained from participants via the Informed Voluntary Participation Form. Participation was entirely voluntary; participants’ identifying information was kept confidential, and the data were anonymized before analysis. For participants under the age of 18, parental/guardian consent and a participant assent (voluntary participation) form were obtained.

### Data collection

The research data were collected in two phases in accordance with the explanatory sequential mixed-method design. After obtaining the necessary institutional permissions, the quantitative data were collected in June–July 2022, and the qualitative data were obtained in November 2022. Participants were reached through club and national team coaches, and participation was based on voluntariness. Quantitative data were collected through online and face-to-face administration, and completion of all scales took approximately 10–12 minutes. Qualitative data were obtained through semi-structured interviews; with participants’ consent, the interviews were audio-recorded and lasted 30–45 minutes. The recordings were transcribed, and all data were prepared for analysis.

### Data analysis

In line with the mixed-method design of the study, the data were analyzed in two phases: quantitative and qualitative. In the quantitative analyses, the measurement models of the scales and the proposed structural model were tested; in the qualitative analyses, interview data were thematically analyzed to deepen the quantitative findings.

### Quantitative data analysis

IBM SPSS Statistics 23 and AMOS 23 software were used to analyze the quantitative data. Prior to the analyses, the dataset was examined in terms of missing data, outliers, normality, and multicollinearity assumptions. Outliers were assessed using z-scores at the univariate level and Mahalanobis distance at the multivariate level. The normality assumption was tested using skewness and kurtosis coefficients, while multicollinearity was examined through tolerance and VIF values [53,54].

The measurement structures of the scales were tested using confirmatory factor analysis, and items with low factor loadings were evaluated by considering theoretical appropriateness and statistical criteria together. Items with standardized factor loadings below.40 were excluded from the analyses [53]. In structural equation modeling, the Maximum Likelihood estimation method was used, and model fit was evaluated using the χ²/df, RMSEA, CFI, TLI, GFI, and AGFI fit indices [55,53]. The direct and indirect structural associations in the research model were tested using AMOS 23, and the significance of indirect associations was examined using bias-corrected bootstrap confidence intervals based on 5,000 bootstrap samples at the 95% confidence level [56].

### Qualitative data analysis

Qualitative data were evaluated using a combination of content analysis and descriptive analysis techniques. After the interview recordings were transcribed into written text, the transcripts were read repeatedly; within the scope of content analysis, meaningful statements were coded, and similar codes were clustered to form categories and themes. Within the scope of descriptive analysis, these themes were organized in line with the research questions and were presented with support from direct quotations. The analysis process was conducted using MAXQDA 2020 software. In this study, the criteria of credibility, transferability, dependability, and confirmability were taken as the basis [57]. To protect participants’ confidentiality, each participant was coded as P1, P2, …, and direct quotations were reported using these codes. To assess coding reliability, interrater agreement between two independent coders was calculated using Cohen’s Kappa coefficient (κ = .93) and was found to indicate an excellent level of agreement according to the classification of Landis and Koch [58].

## Results

In this section, findings related to the measurement model and the structural model from the quantitative phase, as well as the themes derived from the interview data in the qualitative phase, are reported.

### Quantitative findings

In the quantitative phase of the study, data obtained from 374 elite wrestlers were evaluated within the scope of confirmatory factor analysis and structural equation modeling. The quantitative findings report the validity and reliability values of the measurement models, the fit indices of the structural model, the proportions of explained variance, and the standardized direct, indirect, and total structural associations among the variables.

### Testing the measurement model and final structure

The model tested in the study was constructed to examine the structural associations among motivational determinants, enjoyment, and the intention to continue in sport among elite wrestlers. The initial measurement model consisted of 52 items and 13 factors. In this model, goal orientation was represented by task and ego orientations; satisfaction of basic psychological needs was represented by competence, autonomy, and relatedness; and motivation, enjoyment, and intention to continue in sport were represented in line with their respective scale structures. As a result of the confirmatory factor analysis, items with factor loadings below.40 and those that weakened model fit indices were excluded from the analysis.

In order to preserve the theoretical integrity of the model and reduce unnecessary complexity, subdimensions within multidimensional constructs were represented under the relevant second-order/higher-order latent variables. This approach enabled the development of a more parsimonious and interpretable structure while maintaining the explanatory power of the model [55,53,59]. Following these revisions, the final measurement model consisted of 42 items and 8 factors. Item removal decisions were based not only on statistical criteria but also on the extent to which the retained items theoretically represented the relevant latent constructs. Therefore, the final measurement model was evaluated by considering both model fit and the conceptual consistency of the retained items with the relevant constructs. Table 1 presents the distributional characteristics, validity and reliability coefficients, and correlations among the scales. The skewness and kurtosis values were within the commonly accepted thresholds of |skewness| < 3 and |kurtosis| < 10, indicating no serious violation of univariate normality [55]. In addition, the CR, AVE, MSV, ASV, α, and ω values generally supported the convergent validity, discriminant validity, and internal consistency reliability of the measurement model [53].

**Table 1:** Distributional characteristics, validity, reliability, and correlation coefficients for the scales.

Scale	Item	Skew.	Kurt.	CR	AVE	α	ω	MSV	ASV	2	3	4	5	6	7	8
**1. Task Orientation**	5	−1.89	3.35	.86	.55	.84	.85	.27	.16	.35	.37	.30	.36	.37	.34	.18
**2. Ego Orientation**	5	−.987	.804	.83	.50	.81	.83	.27	.09	–	.20	.22	.06	.28	.19	.18
**3. Competence**	5	−.794	−.191	.84	.51	.81	.82	.18	.12		–	.57	.44	.33	.45	.32
**4. Autonomy**	4	−.885	.441	.83	.56	.83	.85	.45	.23			–	.35	.31	.33	.28
**5. Relatedness**	5	−1.20	.835	.89	.62	.88	.89	.45	.16				–	.27	.31	.22
**6. Motivation**	9	−.225	−1.05	.89	.50	.85	.87	.17	.11					–	.36	.43
**7. Enjoyment**	5	−1.43	1.30	.90	.67	.90	.91	.28	.15						–	.30
**8. Intention**	4	−.904	.187	.74	.46	.62	.64	.27	.16							–

**Note.** Skew. = skewness; Kurt. = kurtosis; CR = composite reliability; AVE = average variance extracted; α = Cronbach’s alpha; ω = McDonald’s omega; MSV = maximum shared variance; ASV = average shared variance. Numbered columns 2–8 represent the Pearson correlations among the corresponding scales listed in the first column; “–” indicates the diagonal.

### Structural model and path analysis

Structural equation modeling was conducted to test the proposed research model. First, the measurement structures of the latent variables were evaluated through confirmatory factor analysis, and then the direct and indirect structural associations among the variables were tested within the structural model. Standardized path coefficients (β), significance levels, and model fit indices were taken into account in the analyses.

**Fig 2:**
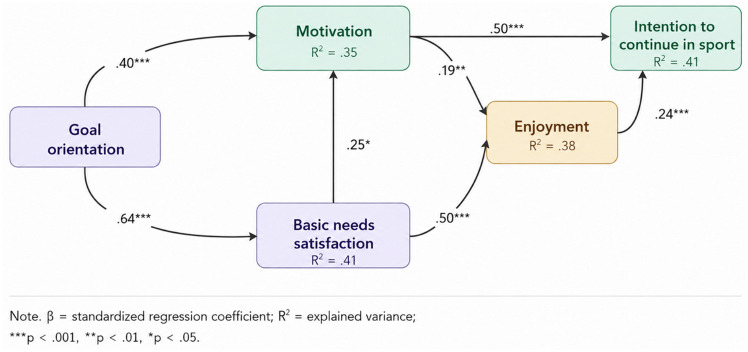
Results of the analyses for the tested structural model.

The overall model fit indices were at an acceptable level: χ²/df = 1.859, GFI = .839, CFI = .907, TLI = .900, NFI = .819, AGFI = .818, RMSEA = .048, and SRMR = .062. These values indicate that the model demonstrated an acceptable fit to the observed data and that the proposed theoretical structure was supported [53]. The path analysis of the structural model is presented in Fig 2, and the full standardized structural equation model with measurement indicators is provided in S4 File. The structural associations among the latent variables in the model, the explained variance values (R²), and significance levels are summarized in Table 2.

**Table 2:** Coefficients and significance levels among variables.

Hypothesis	DV	IV	β₀ (Std.)	β₁ (Unstd.)	R²	S.E.	C.R. (t)	p	Result
**H**_**1**_	BPN	Goal Orientation	0.638	0.451	.407	0.078	5.783	<.001	Accepted
**H**_**2**_	Motivation	Goal Orientation	0.401	0.557	.353	0.160	3.474	<.001	Accepted
**H**_**3**_	Motivation	BPN	0.252	0.494	.353	0.200	2.471	.013	Accepted
**H**_**4**_	Enjoyment	BPN	0.501	1.184	.381	0.194	6.094	<.001	Accepted
**H**_**5**_	Enjoyment	Motivation	0.187	0.225	.381	0.080	2.827	.005	Accepted
**H**_**6**_	Intention	Motivation	0.502	0.401	.414	0.076	5.279	<.001	Accepted
**H**_**7**_	Intention	Enjoyment	0.238	0.158	.414	0.046	3.419	<.001	Accepted

**Note.** DV = dependent variable; IV = independent variable; β = standardized coefficient; B = unstandardized coefficient; S.E. = standard error; C.R. = critical ratio.

According to the tested structural model, Table 2 presents the standardized (β) and unstandardized coefficients for the direct structural associations among the latent variables, along with the explained variance (R²), standard error (S.E.), critical ratio values, and significance levels (p). The results indicate that the hypothesized structural associations among the variables in the model are statistically significant and that the proposed hypotheses (H1–H7) are supported.

### Findings related to the tested hypotheses

According to the findings presented in Table 2, all direct structural associations proposed in the research model were statistically significant, and hypotheses H1–H7 were supported. As shown in Fig 2, goal orientation was positively associated with basic psychological need satisfaction (β = .638, p < .001), explaining 40.7% of the variance in this variable. Goal orientation was also positively associated with motivation (β = .401, p < .001). In addition, basic psychological need satisfaction was positively associated with motivation (β = .252, p = .013) and enjoyment (β = .501, p < .001).

Motivation was positively associated with enjoyment (β = .187, p = .005) and intention to continue in sport (β = .502, p < .001). Enjoyment was also positively associated with intention to continue in sport (β = .238, p < .001). The explained variance (R²) was 35.3% for motivation, 38.1% for enjoyment, and 41.4% for intention to continue in sport. Overall, these findings support the structural associations proposed in the model.

### Direct, indirect, and total effects in the structural equation model

After confirming that the model fit was at an acceptable level, the standardized direct, indirect, and total effects reported in the AMOS output were calculated and are presented in Table 3. Considering the cross-sectional design of the study, these coefficients were interpreted as structural model-based associations rather than causal effects.

**Table 3:** Direct, indirect, and total standardized path coefficients in the structural model.

	Standardized Direct Effects				Standardized Indirect Effects				Standardized Total Effects			
	**GOS**	**BPN**	**MOT**	**ENJ**	**GOS**	**BPN**	**MOT**	**ENJ**	**GOS**	**BPN**	**MOT**	**ENJ**
**BPN**	.638	–	–	–	–	–	–	–	.638	–	–	–
**MOT**	.401	.252	–	–	.161	–	–	–	.562	.252	–	–
**ENJ**	–	.501	.187	–	.424	.047	–	–	.424	.548	.187	–
**INT**	–	–	.502	.238	.383	.257	.044	–	.383	.257	.546	.238

**Note.** GOS = goal orientation in sport; BPN = basic psychological needs satisfaction; MOT = motivation; ENJ = enjoyment; INT = intention to continue in sport.

Table 3 presents the standardized direct, indirect, and total path coefficients among the variables in the structural model. According to the findings, goal orientation was directly and positively associated with basic psychological need satisfaction (β = .638). Its direct association with motivation was β = .401, and its indirect association through basic psychological need satisfaction was β = .161. Therefore, the total association between goal orientation and motivation was β = .562. The association between goal orientation and enjoyment emerged through indirect pathways (β = .424), and its indirect association with intention to continue in sport was β = .383.

Basic psychological need satisfaction was directly and positively associated with motivation (β = .252). Its direct association with enjoyment was β = .501, its indirect association was β = .047, and its total association was β = .548. The association between basic psychological need satisfaction and intention to continue in sport emerged indirectly and was β = .257. Motivation was directly and positively associated with enjoyment (β = .187). Regarding intention to continue in sport, the direct association of motivation was β = .502, the indirect association via enjoyment was β = .044, and the total association was β = .546. Finally, enjoyment was directly and positively associated with intention to continue in sport (β = .238). Overall, the findings indicate that the proposed direct and indirect structural associations in the model were supported.

**Total variance explained by the model:** The model explained approximately 41% of the total variance in intention to continue in sport. This result indicates that the proposed structural model had an adequate level of explanatory power for understanding elite wrestlers’ intention to continue in sport.

Table 4 presents the standardized indirect associations, bias-corrected (BC) bootstrap 95% confidence intervals, and proportion mediated values in the structural model. The findings showed that the indirect association between goal orientation and motivation was borderline but not statistically significant (β indirect = .161, 95% BC CI [−.006,.326], p = .054). In contrast, the indirect associations of goal orientation with enjoyment (β indirect = .424, 95% BC CI [.300,.553], p < .001) and intention to continue in sport (β indirect = .383, 95% BC CI [.272,.512], p < .001) were significant. The indirect associations of basic psychological need satisfaction with enjoyment (β indirect = .047, 95% BC CI [.002,.120], p = .040) and intention to continue in sport (β indirect = .257, 95% BC CI [.080,.414], p = .009) were also significant. In addition, motivation had a significant indirect association with intention to continue in sport through enjoyment (β indirect = .044, 95% BC CI [.009,.119], p = .014). The proportion mediated values indicated that 28.6% of the total association between goal orientation and motivation occurred through basic psychological need satisfaction, whereas 8.1% of the total association between motivation and intention to continue in sport occurred through enjoyment.

**Table 4:** Standardized indirect effects, bootstrap confidence intervals, and proportion mediated.

Predictor	Outcome	β indirect	SE	95% BC CI	p	Proportion mediated
GOS	MOT	.161	.085	[-.006,.326]	.054	28.6%
GOS	ENJ	.424	.064	[.300,.553]	<.001	100%
GOS	INT	.383	.061	[.272,.512]	<.001	100%
BPN	ENJ	.047	.029	[.002,.120]	.040	8.6%
BPN	INT	.257	.085	[.080,.414]	.009	100%
MOT	INT	.044	.026	[.009,.119]	.014	8.1%

**Note.** GOS = goal orientation in sport; BPN = basic psychological needs satisfaction; MOT = motivation; ENJ = enjoyment; INT = intention to continue in sport. β indirect = standardized indirect association; SE = standard error; BC = bias-corrected; CI = confidence interval. Indirect associations were tested using bias-corrected bootstrap 95% confidence intervals. Proportion mediated = standardized indirect association/ standardized total association.

### Analysis of the qualitative findings

In the qualitative phase, open-ended opinions were obtained from 16 elite wrestlers regarding the factors associated with their intention to continue in sport. The data were analyzed using content analysis and descriptive analysis; participants’ statements were coded and structured as themes, categories, and codes. The findings were supported with participant codes (P1–P16), frequency values, and direct quotations. The themes, categories, codes, participants, and frequency values related to the positive and negative factors associated with the intention to continue in sport are presented in Table 5.

**Table 5:** Participants’ views on positive and negative factors associated with the intention to continue in sport.

Theme	Category		Code	Participants	f
Factors Associated with the Intention to Continue in Sport	Positive Factors	Internal	Commitment and passion	P1–P16	16
			Achievement and career goals	P1–P16	16
			Desire and interest	P1–P16	16
			Enjoyment	P1–P16	16
			National pride	P1–P16	16
		External	Reference groups	P1–P16	16
			Coach–athlete interaction	P1–P16	16
			Privileges granted to national athletes	P1–P16	16
			Social influence	P2, P4, P5, P6, P9, P10, P11, P13, P14, P16	10
			Social support	P1–P16	16
	Negative Factors	Internal	Failure	P1–P16	16
			Negative psychological processes	P1–P16	16
		External	Injustice and favoritism	P2, P3, P6, P7, P8, P9, P12, P16	8
			Sport-specific challenges	P1–P16	16
			Lack of resources and support	P1–P16	16

### Theme: Factors associated with the intention to continue in sport

**Fig 3:**
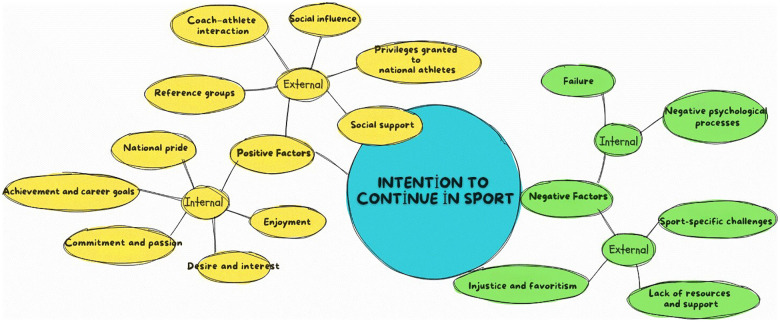
Themes, categories, and codes related to elite athletes’ intention to continue in sport.

As shown in Table 5, the factors associated with participants’ intention to continue in sport were grouped under the theme “Factors Associated with the Intention to Continue in Sport.” This theme was divided into two main categories—positive and negative factors—and each category was further divided into “Internal” and “External” subcategories. The structural relationship among the theme, categories, subcategories, and codes is presented in Fig 3.

### Category 1: Positive factors

The category of positive factors consists of internal and external factors that support elite wrestlers’ intention to continue in sport. Internal factors are related to commitment and passion, achievement and career goals, desire and interest, enjoyment, and national pride. External factors include reference groups, coach–athlete interaction, privileges granted to national athletes, social influence, and social support.

#### Subcategory 1.1: Positive internal factors.

Positive internal factors encompass psychological, emotional, and cognitive factors that support wrestlers’ intention to continue in sport at the individual level. Within this subcategory, five codes were identified: Commitment and passion, Achievement and career goals, Desire and interest, Enjoyment, and National pride. The qualitative data suggest that these codes cluster around the personal meanings athletes attribute to wrestling, their goals, and their positive emotional experiences.

**Commitment and passion (16/16).** All participants expressed a strong commitment to wrestling and a desire to remain in the sport under this code. One participant stated that interest in wrestling gradually turned into commitment over time: *“When I first started wrestling, I began because my family directed me; however, later I gradually started to love this sport and became increasingly attached to it.”* **(P1).** Similarly, another participant emphasized the difficulty of disengaging from the sport and the tendency to return to wrestling: *“Sometimes I quit, but a few days later I found myself back in the gym. It feels like there’s a strange bond between me and this sport.”* **(P5).**

**Achievement and career goals (16/16).** All participants reported that achievement- and career-related goals were associated with their intention to continue in sport. These goals centered on aims such as being selected for the national team, representing the country, and achieving success at the international level. One participant highlighted these goals by stating, *“I set goals for myself to represent my state, my nation, my family, and my country.”* **(P4).** Similarly, another participant noted that goals sustain the decision to remain in sport: *“I still have goals. I continue for the goals I keep inside.”* **(P10).**

**Desire and interest (16/16).** All participants stated that personal interest in and desire for wrestling were associated with their intention to continue in sport. The interviews revealed that for some athletes, orientation toward wrestling began with an intrinsic desire independent of family or environmental influences, whereas for others it became salient through teacher guidance. One participant emphasized this intrinsic interest by stating, *“No one in my family did this sport, but I had love and desire for it inside me.”* **(P5).** Similarly, another participant noted that personal interest and desire were influential in becoming involved in the sport: *“I had an interest and desire for this sport… my teachers noticed my talent.”* **(P11).**

**Enjoyment (16/16).** All participants reported that the enjoyment they derived from wrestling was associated with their intention to continue in sport. One participant indicated that enjoyment helped make difficulties more tolerable by stating, *“I enjoy doing this sport; otherwise, it wouldn’t be worth enduring so many difficulties.”* **(P2).** Similarly, another participant emphasized that pleasure and affection for wrestling were central to the decision to continue: “*I continue with this sport because I truly love it and find joy in it.”* **(P7).**

**National pride (16/16).** All participants stated that a sense of national belonging and the desire to represent their country were associated with their intention to continue in sport. The narratives highlighted that the aspiration to represent the Turkish flag on international platforms and to have the national anthem played constitutes a strong source of motivation for wrestlers. One participant emphasized the desire to represent the Turkish flag internationally by stating, *“I want to carry this flag to the very end and show it to the whole world.”* **(P4).** Similarly, another participant highlighted the importance of national representation in motivation to continue in sport: *“It’s my greatest wish to raise our flag and hear our national anthem played.”* **(P6).**

#### 1.2: Positive external factors.

Positive external factors include social, environmental, and institutional elements associated with wrestlers’ intention to continue in sport. Within this subcategory, five codes were identified: Reference groups, Coach–athlete interaction, Privileges granted to national athletes, Social influence, and Social support. The qualitative data suggest that support mechanisms involving family, coaches, friends, the community, clubs, and institutional structures were related to athletes’ decisions to continue wrestling.

**Reference groups (16/16).** All participants stated that reference groups such as family members, teachers, coaches, and close social circles were associated with both initiating wrestling and sustaining participation. The interviews revealed that, in particular, family members and teachers were related to the intention to continue in sport by recognizing athletes’ potential, providing guidance, and offering encouragement. One participant emphasized the influence of family members on becoming involved in wrestling by stating, *“My older brother and uncle were involved in wrestling. I wanted to be like them, and they guided me.”* **(P1).** Similarly, the statement *“My physical education teacher noticed my talent and encouraged me.”* **(P11)** highlights the role of teacher guidance in the process of sport participation.

**Coach–athlete interaction (16/16).** All participants reported that their interaction with their coaches was associated with their intention to continue in sport. Under this code, athletes noted that the support, attention, and guidance received from coaches were related to their decision to remain in sport and to reduced thoughts of quitting. For example, one participant emphasized this support by stating, *“My coach always took me by the hand and brought me back to wrestling. No matter how much I considered quitting, I continued thanks to his support.”* **(P12).** Similarly, another participant highlighted the coach’s guiding role by stating, *“I regained my lost confidence through my coach. His support and guidance helped me return to the sport.”* **(P14).**

**Privileges granted to national athletes (16/16).** All participants stated that academic, professional, and economic opportunities associated with national athlete status were associated with their intention to continue in sport. Within this code, participants reported that advantages such as scholarships, educational/university opportunities, and employment opportunities were related to their decisions to sustain their sport careers. For example, one participant emphasized scholarship support by stating, *“I gained so much thanks to this sport. Through my success in wrestling, I received a national athlete scholarship throughout my university studies.”* **(P5).** Similarly, another participant highlighted the professional advantage provided by national athlete status by stating, *“Thanks to my achievements, I earned the right to be appointed as a teacher through the national athlete quota.”* **(P7).**

**Social influence (10/16).** Most participants reported that appreciation, respect, and attention from the community were associated with their intention to continue in sport. The narratives highlighted that achievements attained through wrestling were related to recognition and social prestige within athletes’ social environments, which in turn contributed to athletes’ feeling valued and committed to the sport. One participant underscored the social prestige associated with wrestling by stating, *“Thanks to my achievements in wrestling, I gained respect in society.”* **(P2).** Similarly, another participant emphasized the impact of positive feedback from the social environment by stating, *“Because of this sport, I became a person who is loved and respected by my community.”* **(P14).**

**Social support (16/16).** All participants stated that the support they received from family members, coaches, friends, clubs, and/or public institutions was associated with their intention to continue in sport. Within this code, athletes emphasized that emotional support and financial/institutional contributions, in particular, were related to their decision to remain in the sport. For example, one participant highlighted the importance of family support by stating, *“My family’s support is very important to me. Whether I win or lose, they are always by my side. They’ve never left me alone and have always supported me.”* **(P1).** Similarly, another participant drew attention to the importance of institutional support by stating, *“Without the scholarships, awards, and appointment rights provided by our state and sports clubs, it would be very difficult to continue this sport.”* **(P10).**

### Category 2: Negative Factors

The category of negative factors consists of internal and external factors associated with lower levels of elite wrestlers’ intention to continue in sport. The qualitative data suggest that these factors were related to individuals’ psychological and emotional strains as well as environmental, structural, and social barriers. Within this scope, two subcategories were identified: Internal Negative Factors and External Negative Factors. Both subcategories represent key elements that may be associated with a decline in athletes’ intention to continue in sport, loss of motivation, and burnout.

#### Subcategory 2.1: Negative internal factors.

Negative internal factors refer to individual-based difficulties associated with wrestlers’ reduced desire to continue in sport. The findings within this subcategory suggest that athletes sometimes struggle to sustain participation due to experiences of failure, the perception of not meeting expectations, decreased motivation, and negative affective states. Within this scope, two codes were identified: Failure and Negative psychological processes.

**Failure (16/16).** All participants reported that experiences of failure—such as not reaching the expected performance level, not achieving a placing in competitions, or not attaining the targeted improvement—were associated with lower intention to continue in sport. The qualitative data suggest that the perception of failure may be linked to decreased self-confidence, lower motivation, and thoughts of withdrawing from the sport. One participant emphasized that failure negatively shapes one’s perspective toward sport by stating, *“You train intensely, but when success does not come, it both increases your exhaustion and negatively affects how you view the sport; there were periods when I did not want to continue and even considered quitting.”* **(P1).** Similarly, another participant noted that failure experiences can trigger thoughts of withdrawal: *“There were times when I couldn’t win medals in the junior categories; when I thought I couldn’t reach my goal, I considered quitting wrestling.”* **(P3).**

#### Negative psychological processes (16/16).

All participants noted that psychological processes such as stress, anxiety, burnout, decreased motivation, and emotional fatigue were associated with lower intention to continue in sport. The qualitative data suggest that intensive training schedules, high performance expectations, medal pressure, and environmental demands may contribute to psychological strain. One participant emphasized the impact of competitive pressure: *“Competition stress and the medal pressure on me affect me negatively.”* **(P7).** Similarly, another participant described how environmental expectations can make continuation more challenging: *“Expectations from my family and people around me create pressure. This stress sometimes makes it hard for me to continue wrestling.”* **(P16).**

#### Subcategory 2.2: Negative external factors.

Negative external factors include environmental, structural, and systemic factors associated with lower levels of wrestlers’ intention to continue in sport. The qualitative data suggest that perceptions of injustice in the sport environment, sport-specific challenges, and insufficiencies in resources and support may be related to difficulties in sustaining participation. Within this subcategory, three codes were identified: injustice and favoritism, sport-specific challenges, and lack of resources and support.

**Injustice and favoritism (8/16).** Some participants reported experiencing injustice/favoritism in evaluation and selection processes, which may be associated with lower levels of their intention to continue in sport. Within this code, athletes noted that they could encounter unfair practices particularly in areas such as national team trials, training camp rosters, referees’ decisions, and the distribution of financial support. For example, one participant stated, *“Although I qualified for the Olympic quota tournament, not being sent made me consider quitting the sport.”* **(P7).** Similarly, another participant emphasized that damage to the sense of fairness may influence their perspective on the sport: *“Incorrect or biased decisions by referees and coaches… wear me down even more and change my perspective on this sport.”* **(P16).**

**Sport-specific challenges (16/16).** All participants indicated that the high physical and organizational demands of wrestling (intensive training loads, weight control, risk of injury, long training camp periods, and sacrifices in social life) can make sustaining their intention to continue in sport more difficult. Within this code, some athletes reported that weight control and high training loads create a marked pressure. For example, one participant stated, *“Losing weight doesn’t affect me much, but a friend of mine who had to lose 7–8 kilos really struggled… I even have friends who quit the sport because of it.”* **(P2)**, describing the impact of weight cutting. Similarly, another participant noted that the intensive training schedule may make sustaining participation more challenging: *“Sometimes we train three times a day… sometimes the training gets so intense that I feel like I can’t handle it.”* **(P5).**

**Lack of resources and support (16/16).** All participants indicated that structural, institutional, and economic resource limitations may make sustaining their intention to continue in sport more challenging. Participant statements suggest that limited financial support, lack of sponsorship, clubs’ low-salary policies, the small number of clubs providing regular income, and limited support from the federation can create difficulties in maintaining a wrestling career. One participant emphasized the link between insufficient support and consideration of leaving the sport by stating, *“Neither the federation, nor sponsors, nor clubs provide adequate support. There are hardly any clubs that pay a proper salary. Therefore, I want to finish my education, establish my profession, and leave this sport.”* **(P6).** Similarly, another participant highlighted the importance of institutional and economic support in sustaining participation: *“There are very few wrestling clubs in Turkey. Only two or three provide decent salaries. If it weren’t for TOHM and the national athlete scholarships provided by the state, many athletes wouldn’t be able to continue this sport. A lot of my friends quit wrestling because of this.”* **(P8).**

### Integration of quantitative and qualitative findings

The quantitative findings indicated that goal orientation, satisfaction of basic psychological needs, motivation, and enjoyment are associated with the intention to continue in sport. The qualitative findings, in turn, revealed how these relationships are reflected in participants’ experiences. The codes of Commitment and passion, Achievement and career goals, Desire and interest, Enjoyment, Social support, and Coach–athlete interaction showed that the intention to continue in sport is related to both internal and external factors. In particular, the fact that the Enjoyment code was emphasized by all participants was consistent with the significant association between enjoyment and the intention to continue in sport in the quantitative model. In contrast, factors such as Failure, Negative psychological processes, Sport-specific challenges, Injustice and favoritism, and Lack of resources and support were associated with lower levels of intention to continue in sport. When both data types are considered together, the core constructs in the research model appear to be supported in a consistent and complementary manner across different data sources. This holistic view suggests that the mixed-method approach enhances the explanatory depth of the findings and contributes to a more comprehensive understanding of the multidimensional nature of the intention to continue in sport.

## Discussion, conclusion and recommendations

In this study, the motivational determinants associated with elite wrestlers’ intention to continue in sport were examined by jointly evaluating quantitative and qualitative data. All tested hypotheses were found to be statistically significant and were supported. The findings revealed that goal orientation, satisfaction of basic psychological needs, motivation, and enjoyment are important constructs within the model for understanding intention to continue in sport. The qualitative findings also provided explanations that supported and deepened these associations in line with athletes’ experiences. In the discussion section, the quantitative and qualitative findings were addressed with reference to the relevant literature, and similarities, differences, and possible explanations across the findings were evaluated.

### Goal Orientation and Basic Psychological Need Satisfaction (H1)

The analysis results showed that goal orientation was positively associated with basic psychological need satisfaction (β = .638, p < .001). This finding indicates that elite wrestlers’ achievement goal perceptions are associated with the satisfaction of their needs for autonomy, competence, and relatedness. Similarly, previous studies have reported significant relationships between goal orientation and basic psychological need satisfaction [60–62]. This result is consistent with the assumptions of Self-Determination Theory, which posits that psychological need satisfaction is closely linked to motivational processes [15]. In this context, goal orientation can be considered an important motivational construct related to psychological need satisfaction among elite wrestlers.

### Goal Orientation and Motivation (H2)

The analysis results indicated that goal orientation was positively associated with motivation (β = .401, p < .001). This finding suggests that elite wrestlers’ achievement goal perceptions are related to their motivational processes. Likewise, previous studies have shown that goal orientation is associated with athletes’ motivational processes [63,64]. In addition, it has been suggested that achievement goals are linked to motivational regulations in sport contexts and that goal orientation may be related to self-regulatory motivational processes [65,66]. This result aligns with the assumptions of Achievement Goal Theory, which proposes that the meaning individuals attribute to success is related to their motivational orientations [8]. Accordingly, goal orientation may be considered a key construct for understanding motivational processes among elite wrestlers.

### Basic Psychological Need Satisfaction and Motivation (H3)

In this study, a significant and positive relationship was found between basic psychological need satisfaction and motivation among elite wrestlers (β = .252, p = .013). This finding indicates that the satisfaction of elite wrestlers’ needs for autonomy, competence, and relatedness is associated with their motivational processes. Similarly, previous studies have shown that basic psychological need satisfaction is closely related to motivational regulations [67,68]. In addition, meta-analytic evidence suggests that amotivation may increase when need satisfaction is low [69]. This result is consistent with the core assumptions of Self-Determination Theory, which posits that psychological need satisfaction is related to motivational processes [15].

### Basic Psychological Need Satisfaction and Enjoyment (H4)

The analysis results indicated that basic psychological need satisfaction was positively associated with enjoyment (β = .501, p < .001). This finding suggests that elite wrestlers’ satisfaction of autonomy, competence, and relatedness needs is linked to enjoyment derived from sport. Likewise, previous studies have reported that perceived competence and social relationships are related to sport enjoyment [70,71]. Moreover, positive relationships between basic psychological need satisfaction and enjoyment have been documented [28]. These findings align with the proposition of Self-Determination Theory that the satisfaction of basic needs is closely related to enjoyment and motivation [15].

### Motivation and Enjoyment (H5)

In this study, motivation was significantly and positively associated with enjoyment among elite wrestlers (β = .187, p = .005). This finding suggests that athletes’ motivation toward sport is related to more positive perceptions of the sport experience and to perceiving the activity as more enjoyable. Self-Determination Theory emphasizes that more autonomous motivational patterns, supported by psychological need satisfaction, are associated with positive affect and sustainable participation; therefore, the motivation–enjoyment association is consistent with theoretical expectations [15]. Indeed, previous studies have shown that indicators of motivation are positively related to enjoyment and positive experiences [28,72]. Similarly, positive relationships between motivation and enjoyment have been reported in different samples [73,74]. In the context of elite wrestling, this result highlights the importance of considering emotional processes, such as enjoyment, together with motivational processes when examining continued participation under intensive training demands and performance pressure.

### Motivation and Intention to Continue in Sport (H6)

In this study, motivation was positively and significantly associated with elite wrestlers’ intention to continue in sport (β = .502, p < .001). In addition, the indirect association between motivation and intention through enjoyment was β = .044, and the total association between motivation and intention was β = .546. This pattern suggests that the intention to remain in sport may be closely linked to motivational processes and that enjoyment can be considered an important indirect pathway in this relationship. Within the framework of Self-Determination Theory, need satisfaction and motivational processes are expected to covary with sustainable participation tendencies [15]. Likewise, it has been shown that intention to continue in sport is closely related to motivational processes [7]. Moreover, it has been noted that autonomous motivation is associated with intention to engage in sport and that motivational factors are important for understanding intention to participate in sport [37,75]. Recent findings also indicate that autonomous motivation is positively associated with the likelihood of continuing sport participation, whereas amotivation is related to a higher risk of disengagement from sport [76].

### Enjoyment and Intention to Continue in Sport (H7)

The analysis results indicated that enjoyment was positively associated with intention to continue in sport (β = .238, p < .001). This finding suggests that the enjoyment elite wrestlers derive from their sport experience is related to their intention to sustain participation. Enjoyment is not limited to momentary pleasure; rather, it can be considered a multidimensional construct related to achievement, development, reaching goals, and the personal meaning attributed to the sport experience [25,26]. In the Sport Commitment Model, enjoyment is also addressed as one of the core constructs associated with sport commitment and persistence [24,27]. Similarly, it has been shown that enjoyment derived from sport is positively associated with sport participation and intention to continue [28,31,77]. Moreover, enjoyment has been emphasized as an important psychological construct in understanding intention to continue in sport [7].

### Discussion of the qualitative findings

The qualitative findings suggest that elite wrestlers’ intention to continue in sport is related not only to individual motivational sources but also to social, environmental, and structural conditions. Participants’ views indicated that, alongside positive internal and external factors associated with the intention to continue in sport, psychological and structural barriers may also be related to lower levels of this intention. In this section, the qualitative findings were discussed in line with the relevant theoretical approaches and previous research.

### Positive ınternal factors

According to the qualitative findings, positive internal factors were organized around the codes of Commitment and passion, Achievement and career goals, Desire and interest, Enjoyment, and National pride. These findings indicate that elite wrestlers’ intention to continue in sport is associated not only with external conditions but also with internal resources such as commitment to sport, personal goals, interest, enjoyment derived from sport, and a sense of national representation.

“Commitment and passion” emerges as one of the key internal resources for understanding wrestlers’ intention to continue in sport. Participants stated that wrestling holds an indispensable place in their lives and that this passion is associated with their intention to continue in sport. This finding is consistent with the Sport Commitment Model [27]. Similarly, previous studies have shown that sport commitment and passion are associated with the intention to continue in sport [40,78,79].

“Achievement and career goals” also stands out as an important internal resource associated with elite wrestlers’ intention to continue in sport. Participants’ statements regarding being selected for the national team, achieving success at national and international levels, and leaving a lasting mark in wrestling indicate that the intention to continue in sport is related to achievement-oriented goals. This finding aligns with the explanations of Achievement Goal Theory suggesting that the meaning individuals attribute to success is related to motivational orientations [8]. Likewise, it has been shown that goal orientation and motivational climate are associated with sport participation and the intention to continue in sport [37,80].

“Desire and interest” emerges as another important internal resource associated with wrestlers’ voluntary participation in sport and their desire to remain in the sport. Participants’ statements that they continue wrestling with enjoyment despite physical and mental challenges indicate that the intention to continue in sport is associated with personal interest and desire. Similar findings have been reported in the literature. It has been noted that sport attitudes are related to desire and interest and that individual desire is related to participation motivation [81,82]. In addition, interest has been identified as an important factor in the decision to engage in sport [83].

In participants’ statements, “enjoyment” appears as one of the core emotional experiences related to the decision to continue wrestling. Participants reported that the enjoyment they derive from training and competitions is associated with their commitment and their decision to remain in the sport. This finding is consistent with the Sport Commitment Model, which considers enjoyment derived from sport as one of the key factors related to sport commitment and persistence [24]. Similarly, it has been shown that enjoyment derived from sport is associated with participation motivation [28]. In addition, various studies have reported that enjoyment levels are significantly associated with physical activity, sport participation, and the intention to continue in sport [25,26,31,52,84].

“National pride” can be considered one of the internal resources that gives meaning to elite wrestlers’ intention to continue in sport. Participant statements indicate that the desire to represent the flag internationally and to have the national anthem played does not solely reflect individual achievement for athletes; it is also associated with feelings of national belonging, responsibility, and pride. This finding is consistent with previous studies indicating that sport is related to national identity and a sense of belonging [85,86]. Similarly, it has been noted that the desire to be selected for the national team is an important source of motivation for athletes [87].

Overall, positive internal factors indicate that elite wrestlers’ intention to continue in sport is associated with personal sources of meaning such as commitment, achievement goals, interest, enjoyment, and national belonging. These findings are consistent with the goal orientation, motivation, and enjoyment variables in the quantitative model and suggest that the intention to continue in sport is related not only to performance goals but also to the emotional and cultural meanings athletes attribute to wrestling.

### Positive external factors

Positive external factors indicate that the social environment and institutional support are closely associated with elite wrestlers’ intention to continue in sport. Guidance from family members, teachers, coaches, and close social circles—as well as social support, recognition, and privileges granted to national athletes—stand out as external resources related to athletes’ decision to continue wrestling.

“Reference groups” indicate that environmental actors such as family members, teachers, coaches, friends, and role-model athletes are associated with elite wrestlers’ processes of becoming involved in sport and sustaining participation. Participant statements show that these individuals assume a guiding and supportive role not only during sport initiation but also throughout the continuation of athletes’ sporting careers. This is consistent with previous studies showing that reference groups are related to sport participation and continuation processes [88,89].

“Coach–athlete interaction” emerges as one of the key external resources associated with wrestlers’ intention to continue in sport. Participant statements indicate that the attention, support, and guidance received from coaches are related to athletes’ decision to remain in the sport, particularly during periods of difficulty or when thoughts of quitting arise. This finding is consistent with similar studies in the literature [30,80,90]. In addition, positive perceptions of coaches’ behavioral styles have been shown to be associated with athletes’ levels of sport commitment [91].

“Privileges granted to national athletes” highlight the importance of institutional and economic support in elite wrestlers’ intention to continue in sport. Participant statements indicate that opportunities associated with national athlete status—such as scholarships, quota/placement opportunities in education, employment opportunities, and financial rewards—are perceived by athletes as forms of support related to sustaining their careers. This finding appears to be consistent with legal and institutional regulations for national athletes [92–95]. Similarly, it has been noted that institutional support provided to athletes is important for the sustainability of sport careers [96]. Accordingly, these privileges may be considered external resources associated with long-term sport participation through athletes’ education, career development, and financial security.

“Social influence” indicates that appreciation, respect, and recognition from wrestlers’ social environments constitute one of the external resources associated with the intention to continue in sport. Participant statements show that achievements attained through wrestling are related to athletes’ visibility and prestige within their social circles, which may also be associated with their commitment to the sport. This finding is consistent with studies suggesting that social status, recognition, and environmental approval are associated with athletes’ motivations to participate in and continue sport [97–99]. In this respect, social influence can be considered one of the external social resources associated with elite wrestlers’ intention to continue in sport.

The “Social support” code indicates that the support athletes receive from sources such as family members, coaches, friends, clubs, and institutions is associated with their intention to continue in sport. Participant statements reveal that, in particular, support from family and coaches is related to athletes’ motivation, psychological resilience, and decisions to remain in sport. The literature also indicates that social support is related to physical activity, sport participation, performance, and well-being [100,101]. Similarly, coach and parental support have been shown to be associated with intention to continue in sport and subjective well-being [102,103].

Overall, the positive external factors indicate that elite wrestlers’ intention to continue in sport is associated not only with individual motivational processes but also with external resources such as the social environment, coach support, social support, recognition, and institutional opportunities. These findings are consistent with the satisfaction of basic psychological needs and motivation variables in the quantitative model and suggest that the intention to continue in sport is associated with supportive social relationships and institutional opportunities.

### Negative ınternal factors

This subcategory addresses the individual, emotional, and cognitive processes that participants reported may make it more difficult to sustain their intention to continue in sport. Within this scope, the codes “Failure” and “Negative psychological processes” were particularly salient. Participant statements indicate that stress, pressure, and emotional fatigue may increase especially during periods when expected performance levels are not achieved; accordingly, motivation may decline, and the decision to remain in sport may be questioned.

“Failure” indicates that the negative emotional and cognitive processes experienced by elite wrestlers when they do not achieve their goals are associated with their intention to continue in sport. Participant statements suggest that experiences of failure may be related to challenges in self-confidence, perceived competence, and motivation, and may at times be linked to thoughts of disengaging from sport. This finding is consistent with Achievement Goal Theory and Self-Efficacy Theory, as both approaches emphasize that perceptions of success/failure are related to individuals’ competence appraisals and behavioral orientation [8,104]. Similarly, it has been reported that experiences of failure are associated with the decision to quit sport and that such negative experiences may be related to lower intention to continue in sport [84,105].

The “Negative psychological processes” code indicates that internal difficulties such as stress, anxiety, burnout, decreased motivation, and emotional fatigue experienced by athletes may make it more difficult to sustain their intention to continue in sport. Participant statements suggest that, in particular, performance pressure, constant competition, and high expectations are related to emotional burden and may at times lead athletes to question their decision to remain in sport. This is consistent with studies demonstrating the relationship between burnout and the tendency to withdraw from sport [106,107]. Similarly, it has been reported that lack of motivation and psychological pressure are associated with decisions to quit sport and may be linked to disengagement processes among athletes [108].

Overall, negative internal factors indicate that elite wrestlers’ intention to continue in sport is associated not only with the motivational and emotional variables included in the model but also with individual psychological processes outside the model, such as perceptions of failure, stress, anxiety, burnout, and decreased motivation. These findings highlight certain psychological risk areas that are not directly represented in the quantitative model and suggest that the intention to continue in sport may be related not only to supportive factors but also to internal barriers that can complicate the decision to remain in sport. In this respect, the qualitative findings contribute to a more holistic understanding of elite wrestlers’ intention to continue in sport.

### Negative external factors

Negative external factors encompass structural, administrative, and environmental conditions that may make it more difficult for elite wrestlers to sustain their intention to continue in sport. In this respect, injustice and favoritism, sport-specific challenges, and lack of resources and support were prominent. Participant statements indicate that unfair practices, intensive training loads, weight control, injury risk, long training camp periods, and insufficient institutional support may be associated with difficulties in sustaining their wrestling careers.

The code “Injustice and favoritism” reflects wrestlers’ perceptions of non-merit-based practices in national team selection, referees’ decisions, and competition processes. Participant statements suggest that such experiences may be associated with a reduced sense of fairness and greater difficulty in maintaining their intention to continue in sport. This finding is consistent with studies highlighting the role of injustice, discrimination, and organizational problems in athlete dropout and disengagement processes [109,110]. Similarly, it has been noted that elite athletes’ experiences of systematic favoritism may be related to negative consequences for their sport lives [111]. It has also been emphasized that refereeing errors and perceptions of favoritism in combat sports may be negatively associated with athletes’ motivation [112].

“Sport-specific challenges” refer to difficulties arising from the high physical, psychological, social, and educational demands of wrestling. Participant statements indicate that intensive training schedules, weight control, injury risk, prolonged training camp periods, sacrifices in social life, and difficulties in maintaining the education–sport balance are associated with external pressures that may be linked to lower intention to continue in sport. Similarly, the literature reports that excessive training and stress can be associated with negative health and performance outcomes [113], that physical demands may be related to the tendency to drop out of sport [73,106], and that dual-career pressure may be associated with psychological burden [114].

The “Lack of resources and support” code refers to the insufficiency of the financial, structural, and institutional supports required for wrestlers to sustain their careers. Participants stated that factors such as limited economic support, the small number of clubs providing regular income, lack of sponsorship, and inadequate institutional support may be negatively associated with their intention to continue in sport. This finding indicates that economic security and institutional support mechanisms are important for athletes to sustain their careers. Indeed, it has been reported that economic insufficiencies may be associated with lower motivation among young athletes, that the lack of social security among amateur athletes may be related to difficulties in decisions to continue sport, and that the lack of financial support may be one of the reasons for quitting sport [115–117].

Overall, negative external factors indicate that perceptions of fairness in the sport environment, sport-specific demands, and the level of institutional support are important for elite wrestlers’ intention to continue in sport. These findings highlight environmental and systemic risk areas that are not directly represented in the quantitative model but may be associated with lower intention to continue in sport. In this respect, the qualitative findings suggest that decisions to remain in sport among elite wrestlers should be evaluated together with a fair, supportive, and sustainable sport ecosystem.

### Integrated evaluation of quantitative and qualitative findings

In this study, the motivational, emotional, social, and contextual processes associated with elite wrestlers’ intention to continue in sport were examined using an explanatory sequential mixed-method approach. The quantitative findings showed that goal orientation, satisfaction of basic psychological needs, motivation, and enjoyment had significant associations with the intention to continue in sport. The model explained 41% of the variance in intention to continue in sport. The qualitative findings illustrated how these associations were reflected in athletes’ lived experiences and indicated that the intention to continue in sport was associated with supportive processes such as commitment and passion, achievement goals, enjoyment, social support, coach–athlete interaction, and national pride. When the quantitative and qualitative findings are considered together, the intention to continue in sport appears to extend beyond the motivational and emotional variables included in the model. Factors emerging from the qualitative findings—such as commitment and passion, national pride, social influence, coach–athlete interaction, and institutional opportunities—were consistent with the associations in the quantitative model at an experiential level. In contrast, themes such as failure, negative psychological processes, injustice and favoritism, sport-specific challenges, and lack of resources and support highlighted internal and external risk areas that are not directly included in the model but may be associated with lower intention to continue in sport.

This integrated evaluation suggests that the associations statistically identified in the quantitative model were further clarified through qualitative data. In other words, while the quantitative analyses identified the associations among the core variables related to the intention to continue in sport, the qualitative findings clarified how these associations are reflected in athletes’ daily experiences through meanings, sources of support, and constraining conditions. In this respect, the study findings are generally consistent with the core assumptions of Achievement Goal Theory, Self-Determination Theory, the Theory of Planned Behavior, and the Sport Commitment Model. In conclusion, elite wrestlers’ intention to continue in sport emerges as a multidimensional construct that should be evaluated not only in terms of psychological variables such as goal orientation, need satisfaction, motivation, and enjoyment, but also in relation to athletes’ personal meaning systems, social relationships, institutional support opportunities, and the structural barriers they encounter. Therefore, practices aimed at supporting sport continuation should focus not only on individual motivational processes but also on creating a fair, supportive, and sustainable sport environment.

### Limitations of the study

This study has several limitations. First, the study group was limited to elite-level wrestlers from Türkiye; therefore, the findings should not be directly generalized to athletes from other countries, different sports, age groups, or performance levels. In addition, the quantitative sample included more men than women; this gender imbalance should be considered when interpreting and generalizing the findings. Because the quantitative data were collected cross-sectionally, the relationships among the variables should be evaluated at an associational rather than causal level. Another limitation concerns the measurement model. The exclusion of some items from the analyses, the marginal reliability coefficients of the intention to continue in sport scale, and the relatively low values of some fit indices require caution when interpreting the findings. The qualitative findings were also limited to the views of 16 elite wrestlers and were based on participants’ personal experiences. Despite these limitations, using quantitative and qualitative data together contributed to a multidimensional understanding of the intention to continue in sport.

### Recommendations

In future research, it is recommended that models explaining the intention to continue in sport incorporate psychosocial and environmental variables such as coach–athlete interaction, social support, passion, sport commitment, perceptions of fairness, and institutional support. In addition, conducting comparative studies across different age groups, genders, performance levels, and sport disciplines may contribute to a more comprehensive understanding of how the intention to continue in sport is shaped in different contexts. The use of longitudinal, experimental, or quasi-experimental research designs may further clarify the temporal nature of the relationships among variables and potential causal mechanisms. From an applied perspective, it is important for coaches to adopt an approach that supports not only technical development but also athletes’ psychological needs, motivation, and enjoyment derived from sport. It is recommended that sport clubs, federations, and sport policy makers establish fair, inclusive, and sustainable sport environments and develop support mechanisms that strengthen athlete well-being, social support, career security, and access to resources. Such arrangements may contribute to supporting sport continuation among elite wrestlers and to making long-term athlete development more sustainable.

## Supporting information

S1 File Dataset. Anonymized quantitative raw dataset underlying the statistical analyses.
(XLSX)

S2 File Dataset. Anonymized SPSS/AMOS dataset with English variable labels.
(SAV)

S3 File Qualitative Dataset. Anonymized qualitative excerpts, keywords, and codes supporting the qualitative findings.
(XLSX)

S4 File Measurement model and standardized structural path model. (DOCX)

S5 File Participant information and informed consent protocol. (DOCX)

S6 File Measurement instruments, retained items, and interview forms used in the study. (DOCX)

S7 File Codebook for demographic variables in the quantitative dataset. (DOCX)
