# Supplementary figures and images for: The effect of motivational determinants on elite Wrestlers’ ıntentions to continue in sport: The mediating role of enjoyment
Source: PLoS One. 2026 Jul 10;21(7):e0353067. doi: 10.1371/journal.pone.0353067 (PMC13353986; doi:10.1371/journal.pone.0353067)

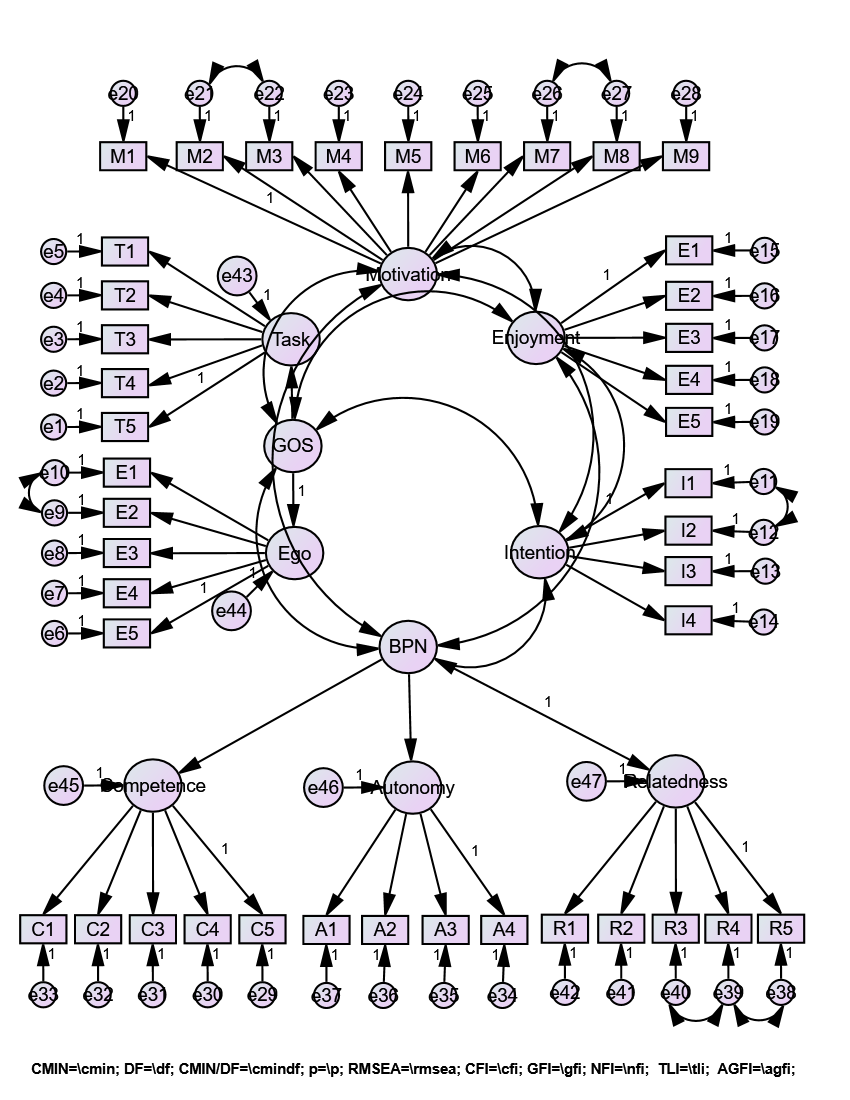


**Standardized Measurement Model Path Diagram**


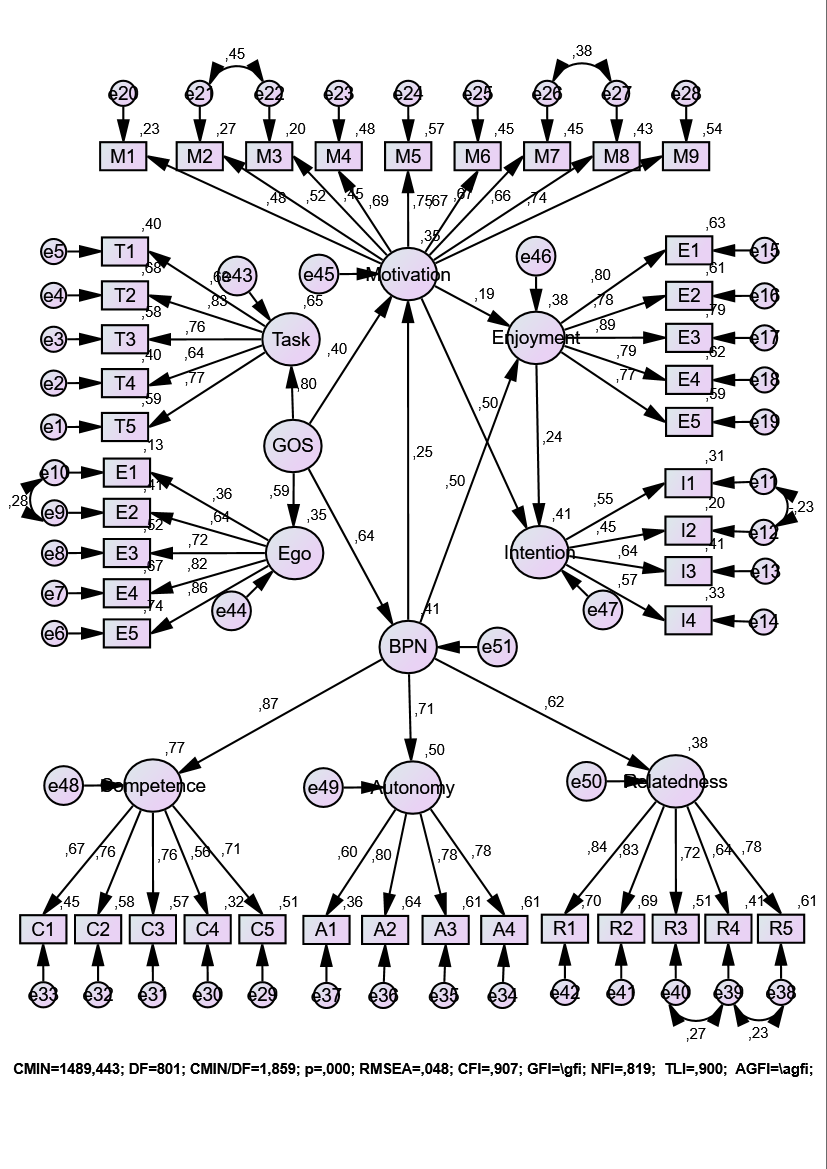


**Standardized Structural Model Path Diagram**

Supplement: S4 File — (DOCX) [file pone.0353067.s004.docx]
