# Supplementary material for: The effect of motivational determinants on elite Wrestlers’ ıntentions to continue in sport: The mediating role of enjoyment
Source: PLoS One. 2026 Jul 10;21(7):e0353067. doi: 10.1371/journal.pone.0353067 (PMC13353986; doi:10.1371/journal.pone.0353067)
Supplement: S5 File — (DOCX) [file pone.0353067.s005.docx]

**INFORMED CONSENT AND PARTICIPANT INFORMATION PROTOCOL**

| **Study Title:** | The Effect of Motivational Determinants on Elite Wrestlers’ Intentions to Continue in Sport: The Mediating Role of Enjoyment |
| --- | --- |
| **Researcher:** | Burhan Özkurt |

**Dear Participant,**

You are invited to participate in a research study entitled “The Effect of Motivational Determinants on Elite Wrestlers’ Intentions to Continue in Sport: The Mediating Role of Enjoyment.”

The purpose of this study is to examine the associations among goal orientation, satisfaction of basic psychological needs, motivation, enjoyment, and elite wrestlers’ intention to continue in sport. The study also aims to gain an in-depth understanding of athletes’ perceptions and experiences regarding the psychological, social, and emotional factors associated with their intention to continue participating in sport.

Participation in the quantitative phase will require approximately 10–12 minutes. Participation in the qualitative interview phase will require approximately 30–45 minutes.

# Voluntary Participation

- Participation in this study is entirely voluntary.
- You will be informed about the purpose and scope of the study, the procedures to be implemented, confidentiality principles, and your right to withdraw.
- You may decline participation or withdraw from the study at any time without providing a reason and without any negative consequences.
- You are encouraged to answer all questions sincerely and freely, without pressure or external influence.
- Signing this form indicates that you have voluntarily agreed to participate in the study.

# Confidentiality and Anonymity

All information obtained during the study will be treated confidentially. Participants’ identifying information will not be disclosed, and the data will be anonymized before analysis. Participant codes such as P1, P2, and P3 will be used instead of names when reporting qualitative findings.

Only anonymized data will be used for scientific analysis and reporting.

# Data Collection Procedures

The study will be conducted in two phases.

## Quantitative Phase

Quantitative data will be collected through online and face-to-face administration of the study scales. Completion of the scales will take approximately 10–12 minutes.

## Qualitative Phase

Qualitative data will be collected through semi-structured interviews. With the participant’s consent, the interviews will be audio-recorded and will last approximately 30–45 minutes. The recordings will be transcribed, anonymized, and prepared for analysis.

**The qualitative data collection process will include:**

- Explanation of the purpose and scope of the study;
- Conducting the semi-structured interview;
- Audio-recording the interview with the participant’s consent;
- Transcribing the interview recording;
- Anonymizing and organizing the data; and
- Analyzing the data through systematic coding.

# Ethical Approval and Data Collection Timeline

The study received ethical approval from the Ethics Committee of the Graduate Education Institute Directorate of Sakarya University of Applied Sciences.

**Ethics approval date:** 21 May 2021

**Approval number:** E-26428519-044-12077

The study was conducted in accordance with ethical principles and the principles of the Declaration of Helsinki.

**Quantitative data collection:** June–July 2022

**Qualitative data collection:** November 2022

# Informed Consent

Written informed consent will be obtained from all participants before data collection.

For participants aged 18 years and older, written informed consent will be obtained directly from the participant.

**For participants aged 17 years:**

- Written assent will be obtained from the participant; and
- Written informed consent will additionally be obtained from a parent or legal guardian.

Participants taking part in the qualitative interviews will also provide consent for audio-recording.

# Participant Group

**The quantitative phase of the study includes 374 elite wrestlers:**

- 325 men and 49 women;
- Aged between 17 and 35 years;
- Actively competing in freestyle or Greco-Roman wrestling;
- Affiliated with the Turkish Wrestling Federation;
- Competing in Junior, U23, or Senior categories; and
- Having national or international competition experience within the previous four years.

**The qualitative phase includes 16 volunteer elite wrestlers:**

- 14 men and 2 women;
- Aged between 20 and 33 years;
- Actively competing in freestyle or Greco-Roman wrestling; and
- Having national or international competitive experience.

# Contact Information

For further information about the study or if you have any questions or concerns, you may contact the researcher:

**Researcher:** Burhan Özkurt

**E-mail:** burhanozkurt@cumhuriyet.edu.tr

**Telephone:** ______________________________

# Participant Consent Statement

**I confirm that:**

- I have read and understood the information provided above.
- I have been informed about the purpose, scope, and procedures of the study.
- I understand that my participation is voluntary.
- I understand that I may withdraw from the study at any time without any negative consequences.
- I understand that my personal information will be kept confidential and that the data will be anonymized.
- I voluntarily agree to participate in this study.

| **Participant’s Name and Surname:** | ____________________________________________ |
| --- | --- |
| **Participant’s Signature:** | ____________________________________________ |
| **Date:** | ________________ |

# For Participants Aged 17 Years

I confirm that I have been informed about the study and voluntarily agree to participate.

| **Participant’s Name and Surname:** | ____________________________________________ |
| --- | --- |
| **Participant Assent Signature:** | ____________________________________________ |
| **Date:** | ________________ |

I confirm that I am the parent or legal guardian of the participant and provide written informed consent for their participation in the study.

| **Parent/Legal Guardian’s Name and Surname:** | ____________________________________________ |
| --- | --- |
| **Parent/Legal Guardian’s Signature:** | ____________________________________________ |
| **Date:** | ________________ |

# Consent for Audio-Recording

I agree that the qualitative interview may be audio-recorded for research purposes.

**☐ Yes ☐ No**

| **Participant’s Name and Surname:** | ____________________________________________ |
| --- | --- |
| **Participant’s Signature:** | ____________________________________________ |
| **Date:** | ________________ |
