# Supplementary material for: The effect of motivational determinants on elite Wrestlers’ ıntentions to continue in sport: The mediating role of enjoyment
Source: PLoS One. 2026 Jul 10;21(7):e0353067. doi: 10.1371/journal.pone.0353067 (PMC13353986; doi:10.1371/journal.pone.0353067)
Supplement: S6 File — (DOCX) [file pone.0353067.s006.docx]

**Measurement Instruments Used in the Study and Items Retained in the Final SEM Model**

**Note.** This document summarizes the demographic information form, the measurement instruments used in the study, the qualitative interview forms, and the specific scale items retained in the final measurement and structural equation model after confirmatory factor analysis. Items not retained in the final SEM model were excluded based on low factor loadings or because they weakened model fit, while also considering theoretical representativeness.

# Summary of retained items in the final SEM model

| **Scale** | **Original number of items** | **Retained items in final SEM model** | **Original item numbers retained** |
| --- | --- | --- | --- |
| Exercise Goal Orientation Scale | 10 | 10 | 1, 2, 3, 4, 5, 6, 7, 8, 9, 10 |
| Basic Psychological Needs Satisfaction in Sport Scale | 14 | 14 | 1, 2, 3, 4, 5, 6, 7, 8, 9, 10, 11, 12, 13, 14 |
| Sport Motivation Scale–II | 16 | 9 | 3, 5, 7, 9, 10, 12, 14, 15, 16 |
| Physical Activity Enjoyment Scale | 8 | 5 | 3, 4, 5, 6, 8 |
| Intention to Continue in Sport Scale | 6 | 4 | 1, 4, 5, 6 |

# Demographic Information Form

| **No.** | **Question / variable** | **Response format** |
| --- | --- | --- |
| 1 | Age | Open-ended numeric value |
| 2 | Gender | Men / Women |
| 3 | Educational background | Primary school / Secondary school / High school / University / Postgraduate |
| 4 | Wrestling style | Freestyle / Greco-Roman |
| 5 | Years engaged in this sport | Open-ended or categorized according to dataset coding |
| 6 | National team level | Senior National (A Team) / Junior National (B Team) / Cadet National (C Team) |
| 7 | Best achievement in wrestling | Open-ended or coded achievement category |
| 8 | Current competition category | Junior / U-23 / Senior |
| 9 | Number of times representing Turkey in international competitions | Open-ended or categorized according to dataset coding |
| 10 | Weekly training frequency | 1–3 times / 4–6 times / 7–9 times / 10 or more times |
| 11 | Weekly training duration | 2–6 hours / 7–10 hours / 11–19 hours / 20 hours or more |
| 12 | Annual competition frequency | 1–3 / 4–7 / 8–10 / 11 or more |

# Exercise Goal Orientation Scale

(Petherick & Markland, 2008; Turkish adaptation by Ersöz et al., 2017)

The scale consists of 10 items rated on a 5-point Likert scale ranging from 1 = Strongly Disagree to 5 = Strongly Agree. All 10 items were retained in the final SEM model.

| **Original item no.** | **Item** | **Subdimension** | **Retained in final SEM model** |
| --- | --- | --- | --- |
| 1 | I feel good when I do my best during exercise. | Task orientation | Yes |
| 2 | I feel good when others cannot exercise as well as I can. | Ego orientation | Yes |
| 3 | I feel good when I make progress in exercise. | Task orientation | Yes |
| 4 | I feel good when I achieve the exercise goals I have set for myself. | Task orientation | Yes |
| 5 | I feel good when I show other exercisers that I am the best. | Ego orientation | Yes |
| 6 | I feel good when I make improvements in exercise. | Task orientation | Yes |
| 7 | I feel good when I prove to myself that I am the only one who can perform a particular exercise. | Ego orientation | Yes |
| 8 | I feel good when I realize that I have more ability than other exercisers. | Ego orientation | Yes |
| 9 | I feel good when I perform at a level that reflects my personal improvement. | Task orientation | Yes |
| 10 | I feel good when I prove to others that I am the best. | Ego orientation | Yes |

# Basic Psychological Needs Satisfaction in Sport Scale

(Ng et al., 2011; Turkish adaptation by Gümüşay & Argan, 2019)

The scale consists of 14 items rated on a 5-point Likert scale ranging from 1 = Strongly Disagree to 5 = Strongly Agree. All 14 items were retained in the final SEM model.

| **Original item no.** | **Item** | **Subdimension** | **Retained in final SEM model** |
| --- | --- | --- | --- |
| 1 | I can overcome challenges related to my sport. | Competence | Yes |
| 2 | I am skilled in my sport. | Competence | Yes |
| 3 | I think I am good at my sport. | Competence | Yes |
| 4 | I have opportunities that make me feel competent in my sport. | Competence | Yes |
| 5 | I have the ability to perform my sport well. | Competence | Yes |
| 6 | I have opportunities to make choices in my sport. | Autonomy | Yes |
| 7 | I have a say in how things are done in my sport. | Autonomy | Yes |
| 8 | I can participate in the decision-making process in my sport. | Autonomy | Yes |
| 9 | I have opportunities to make decisions in my sport. | Autonomy | Yes |
| 10 | I feel close to my teammates in my sport. | Relatedness | Yes |
| 11 | I show interest in my teammates. | Relatedness | Yes |
| 12 | My teammates care about me. | Relatedness | Yes |
| 13 | I have teammates I can trust. | Relatedness | Yes |
| 14 | I develop close relationships with my teammates. | Relatedness | Yes |

# Sport Motivation Scale–II (SMS–II)

(Pelletier et al., 2013; Turkish adaptation by Yıldız et al., 2019)

The original scale consists of 16 items rated on a 7-point Likert scale ranging from 1 = Does not correspond at all to 7 = Corresponds exactly. In the final SEM model, 9 retained items were used to represent the motivation construct. The retained original item numbers were 3, 5, 7, 9, 10, 12, 14, 15, and 16.

| **Original item no.** | **Item** | **Subdimension** | **Retained in final SEM model** |
| --- | --- | --- | --- |
| 1 | Because I would feel bad if I did not take time to practice sport. | Introjected regulation | No |
| 2 | I used to have good reasons for doing sport, but now I ask myself whether I should continue. | Amotivation | No |
| 3 | Because practicing sport reflects who I am. | Integrated regulation | Yes |
| 4 | Because people I care about would be upset with me if I did not practice sport. | External regulation | No |
| 5 | Because I see it as a good way to develop aspects of myself that I value. | Identified regulation | Yes |
| 6 | Because I think others would not approve of me if I did not practice sport. | External regulation | No |
| 7 | Because I find it enjoyable to discover new performance strategies. | Intrinsic regulation | Yes |
| 8 | I do not know why I do it anymore; I think I am not capable of succeeding in the sport I practice. | Amotivation | No |
| 9 | Because participating in sport is an integral part of my life. | Integrated regulation | Yes |
| 10 | Because I have chosen this sport as a way to improve myself. | Identified regulation | Yes |
| 11 | It is no longer clear to me why I practice sport; I do not really think I have a place in sport. | Amotivation | No |
| 12 | Because through sport, I live in accordance with the principles that I value most. | Integrated regulation | Yes |
| 13 | Because people around me reward me when I practice sport. | External regulation | No |
| 14 | Because I feel better when I practice sport. | Introjected regulation | Yes |
| 15 | Because I enjoy learning more about the sport I do. | Intrinsic regulation | Yes |
| 16 | Because it is one of the best ways I have found to develop other aspects of myself. | Identified regulation | Yes |

**Note.** The final SEM model retained SMS items 3, 5, 7, 9, 10, 12, 14, 15, and 16. These retained items represent integrated regulation, identified regulation, intrinsic regulation, and one introjected regulation item. Items not retained were excluded based on low factor loadings or because they weakened model fit, while also considering theoretical representativeness.

# Physical Activity Enjoyment Scale (PACES)

(Mullen et al., 2011; Turkish adaptation by Özkurt et al., 2022)

The original scale consists of 8 items rated on a 7-point Likert scale ranging from 1 = Strongly Disagree to 7 = Strongly Agree. In the final SEM model, 5 retained items were used to represent enjoyment. The retained original item numbers were 3, 4, 5, 6, and 8.

| **Original item no.** | **Item** | **Construct** | **Retained in final SEM model** |
| --- | --- | --- | --- |
| 1 | I find physical activities enjoyable. | Enjoyment | No |
| 2 | Physical activities are very fun. | Enjoyment | No |
| 3 | Physical activities are pleasant. | Enjoyment | Yes |
| 4 | Physical activities are energizing. | Enjoyment | Yes |
| 5 | Physical activities are satisfying. | Enjoyment | Yes |
| 6 | Physical activities are happiness-giving. | Enjoyment | Yes |
| 7 | Physical activities are motivating. | Enjoyment | No |
| 8 | Physical activities are relaxing. | Enjoyment | Yes |

# Intention to Continue in Sport Scale

(Özkurt, 2023; validated by Özkurt et al., 2025)

The original scale consists of 6 items rated on a 7-point Likert scale ranging from 1 = Strongly Disagree to 7 = Strongly Agree. In the final SEM model, 4 retained items were used to represent intention to continue in sport. The retained original item numbers were 1, 4, 5, and 6.

| **Original item no.** | **Item** | **Construct** | **Retained in final SEM model** |
| --- | --- | --- | --- |
| 1 | I will continue this sport in the coming months/years. | Intention to continue in sport | Yes |
| 2 | I often think about quitting this sport. | Intention to continue in sport; reverse-coded in original form | No |
| 3 | I intend to quit this sport. | Intention to continue in sport; reverse-coded in original form | No |
| 4 | Continuing this sport is more of a necessity than a desire for me. | Intention to continue in sport | Yes |
| 5 | It would be very difficult for me to leave this sport. | Intention to continue in sport | Yes |
| 6 | If I decided to leave this sport, many things in my life would be disrupted. | Intention to continue in sport | Yes |

**Note.** The two reverse-worded items in the original scale were not retained in the final SEM model because they had low factor loadings or weakened model fit.

# Draft Interview Form

In the initial stage of the research, a draft interview form consisting of 15 questions related to the psychological processes influencing the intention to continue in sport was developed. Following a content validity evaluation by three field experts, this draft was reduced to six core questions. The six-question draft interview form is presented below.

1. What are your general thoughts and opinions about wrestling as a sport?

2. What were your reasons for starting wrestling?

3. What are the positive or negative processes that influence your decision to continue wrestling?

4. How do you feel while wrestling? What are the positive and negative emotions and feelings you experience?

5. What are your future expectations or goals in this sport? What are the obstacles to achieving these expectations and goals?

6. What are your thoughts and opinions about the effects of wrestling on your life? What would you recommend to those who are new to this sport?

# Final Semi-Structured Interview Form

After expert feedback and pilot interviews, the wording and sequence of the questions were refined. The final version of the interview form consists of the following core question and its associated follow-up (probe) questions.

## Core Question

What are the views of elite wrestlers on the factors influencing their intention to continue in sport?

## Probing Questions

1. What are the factors that influence your intention to continue participating in sports?

2. What are the most significant factors that affect your decision to remain in sports?

3. Could you describe the situations, experiences, or processes that have positively or negatively affected this decision?

# Additional Note

The full texts of the measurement instruments used in this study, as well as the methodological details regarding the qualitative interview questions, are presented in detail in the doctoral dissertation from which this study was derived. The related dissertation is publicly accessible through the National Thesis Center of the Council of Higher Education (YÖK), Türkiye:

https://tez.yok.gov.tr/UlusalTezMerkezi/tezSorguSonucYeni.jsp

The audio recordings of the qualitative interviews have not been uploaded as supplementary files due to ethical and confidentiality reasons. However, anonymized audio recordings or their transcripts can be provided upon request for editorial or reviewer evaluation purposes.
