# Supplementary material for: The effect of motivational determinants on elite Wrestlers’ ıntentions to continue in sport: The mediating role of enjoyment
Source: PLoS One. 2026 Jul 10;21(7):e0353067. doi: 10.1371/journal.pone.0353067 (PMC13353986; doi:10.1371/journal.pone.0353067)
Supplement: S7 File — (DOCX) [file pone.0353067.s007.docx]

**Codebook for Demographic Variables**

*Supplementary file for Figshare / PLOS ONE data documentation*

**Note.** This codebook defines the demographic variables included in the anonymized dataset. IDNo refers to an anonymized participant identification number and does not contain personally identifying information.

**Recommendation.** This file can be uploaded to Figshare as a supporting material together with the anonymized quantitative dataset. It may be titled "Codebook for demographic variables" or "Demographic variables codebook".

| **Variable** | **Label** | **Coding / description** |
| --- | --- | --- |
| IDNo | Anonymized participant identification number | Unique anonymized participant ID |
| D1 | Age | Open-ended numeric value |
| D2 | Body weight | Open-ended numeric value in kilograms |
| D3 | Weight category | Open-ended / reported wrestling weight class |
| D4 | Gender | 1 = Men; 2 = Women |
| D5 | Educational status | 1 = High school; 2 = Associate degree; 3 = Undergraduate; 4 = Graduate |
| D6 | Wrestling style | 1 = Greco-Roman; 2 = Freestyle |
| D7 | Years of sport experience | 1 = 1-3 years; 2 = 4-6 years; 3 = 7-9 years; 4 = 10 years or more |
| D8 | National athlete status | 1 = A national team; 2 = B national team; 3 = C national team |
| D9Senior | Best achievement in senior category | 1 = Turkish Championship; 2 = International tournaments; 3 = Mediterranean Games; 4 = European Championship; 5 = World Championship; 6 = Olympic Games; 7 = No achievement; 9 = Missing |
| D9U23 | Best achievement in U23 category | 1 = Turkish Championship; 2 = International tournaments; 3 = Mediterranean Games; 4 = European Championship; 5 = World Championship; 6 = Olympic Games; 7 = No achievement; 9 = Missing |
| D9Junior | Best achievement in junior category | 1 = Turkish Championship; 2 = International tournaments; 3 = Mediterranean Games; 4 = European Championship; 5 = World Championship; 6 = Olympic Games; 7 = No achievement; 9 = Missing |
| D10 | Current wrestling competition category | 1 = Senior; 2 = U23; 3 = Junior |
| D11 | Number of times representing Turkiye in international competitions | 1 = 1-3 times; 2 = 4-6 times; 3 = 7-9 times; 4 = 10 times or more |
| D12 | Weekly training frequency | 1 = 1-3 sessions; 2 = 4-6 sessions; 3 = 7-9 sessions; 4 = 10 sessions or more |
| D13 | Weekly training duration | 1 = 2-6 hours; 2 = 7-11 hours; 3 = 12-16 hours; 4 = 17-21 hours; 5 = 22 hours or more |
| D14 | Annual competition frequency | 1 = 1-3 competitions; 2 = 4-6 competitions; 3 = 7-9 competitions; 4 = 10 competitions or more |
| D15 | Injury status in the last three years | 1 = Yes; 2 = No |

**Suggested Figshare description:**

This file provides the codebook for the demographic variables included in the anonymized quantitative dataset. It defines each variable name, its English label, and the coding scheme used in the dataset.

**Suggested file name:** Demographic_Variables_Codebook.docx
